# A new index to measure the uniformity of remolded loess

**DOI:** 10.1038/s41598-024-57797-2

**Published:** 2024-03-25

**Authors:** Haike Wang, Ken Howard, Jianbing Peng, Hui Qian, Yanyan Gao, Panpan Xu

**Affiliations:** 1 School of Water and Environment, Chang’an University, Xi’an, 710054 Shaanxi China; 2 Key Laboratory of Subsurface Hydrology and Ecological Effect in Arid Region of Ministry of Education, Chang’an University, Xi’an, 710054 Shaanxi China; 3 Department of Physical and Environmental Sciences, University of Toronto Scarborough, 1265 Military Trail, Toronto, ON M1C 1A4 Canada; 4 College of Geological Engineering and Geomatics, Chang’an University, Xi’an, 710054 Shaanxi China

**Keywords:** Hydrology, Natural hazards

## Abstract

The uniformity of remolded loess is crucial for engineering stability and in laboratory testing, as it affects physical and mechanical properties. It is important to have an index which can accurately and conveniently evaluate the uniformity of remolded loess. This study demonstrated and verified the feasibility of using hydraulic conductivity (*K*) as an indicator for evaluating the uniformity of remolded loess through laboratory experiments and theoretical analysis. In laboratory research, nine loess samples under different preparation conditions were meticulously prepared in duplicate, which were divided into three sets according to the whole dry density (WDD) of approximately 1.3 g/cm^3^, 1.4 g/cm^3^, and 1.5 g/cm^3^ respectively. For the nine duplicate samples, two procedures were performed for each of the sample. One is the uniformity analysis by cutting the soil column and weighing. The other is the hydraulic conductivity experiment. Results showed that sample uniformity is affected by sample preparation conditions, and there are differences in the uniformity of the same WDD samples. The values of *K* positively correlate with the degree of sample uniformity. In theoretical analysis, based on Darcy’s Law and Kozeny-Carman equation, it is found *K* is inversely proportional to the variance ($\sigma^{2}$) of the sample dry density. That is, *K* is positively proportional to the sample uniformity. Since *K* can be easily determined in the laboratory, the application of this new index in the field of geotechnical engineering makes it very convenient and simple to evaluate the uniformity of remolded loess.

## Introduction

Loess is widely distributed in China, Europe, the United States, Argentina and mainly occurs in semiarid and arid regions around the world^1–3^. Among them, the Loess Plateau of China covers an area of 63.5 km^2^, which has the most extensive and thickest loess deposition in the world^4–6^. In recent years, with the implementation of various national and regional strategies to develop the Loess Plateau of China, loess construction projects increased unprecedentedly^1,7–9^. Loess, as an economical and easily accessible construction material, is widely used in engineering constructions such as high-filled foundations, subgrade, and embankments^8,10–12^. These engineering constructions need to compact the loess to improve its strength and reduce its compressibility and permeability.

Compaction uniformity is one of the most crucial factors affecting engineering performance and it has been an important index to evaluate engineering quality and safety^13,14^. During compacting, the degree of compaction (also known as whole dry densities (WDD)) can be carefully monitored and controlled in laboratory^15,16^, but there is no guarantee that the remolded loess is homogeneous. Compacting loess on site, the loess must be compacted to the designed compactness by applied load^13^. It is inevitable that there are over-compaction areas and under-compaction areas due to the limited influence depth of compaction^13,14^. Depending on the sample preparation method adopted, remolding can affect sample uniformity to varying degrees such that dry density may vary significantly throughout the remolded sample^17–21^. Even when identical and consistent compaction techniques are used, remolded loess uniformity can vary due to equipment and operation factors^22^.

Recognizing that it is virtually impossible to obtain completely homogeneous remolded soil samples, at least on a regular basis^23^, it seems logical that an assessment of sample uniformity should be an obligatory pre-requisite for geotechnical testing. Unfortunately, there is no indicator that can directly represent the sample uniformity at present, and existing methods for analyzing sample uniformity are difficult, time-consuming and uneconomic. They also frequently involve the painstaking measurement of multiple parameters. For example, Bellotti, et al.^24^ predicted uniformity of soil samples using determinations of thermal conductivity which vary as a function of dry density. This method needs to embed thermal sensor into soil sample, which is not suitable for small sample and will destroy the integrity of soil sample. Dry density variations in remolded samples have also been analyzed using imaging techniques (X-ray, gamma-ray and optical) by Frost and Park^25^ and Bradshaw and Baxter^26^. However, this mothed is limited by scanning accuracy. The soil sample should be small enough to obtain satisfactory observation results, e.g. when the scanning accuracy reaches 3.25 micron, the diameter of the sample cannot exceed 5 mm^27^. Therefore, the imaging techniques cannot meet the accuracy requirements for the soil sample of laboratory test.

*K* is one of the most important properties in the fields of geotechnical, geological, and civil engineering^28,29^, and it is closely related to the soil dry density. However, the uniformity of soil sample affects the distribution of dry density in the soil sample, so that the difference of sample uniformity will lead to the difference of hydraulic conductivity. Hydraulic conductivity is required to be measured in almost all geotechnical tests. If a relationship between hydraulic conductivity and sample uniformity can be established and verified, the use of hydraulic conductivity as a new index of uniformity becomes feasible. It can not only overcome the shortcomings of previous methods, but also not add too much extra work.

In the paper presented here, a new index is introduced—the property of hydraulic conductivity (*K*)- that can provide a very convenient gauge of sample uniformity. The paper begins by outlining the methodology for preparing, in duplicate, three sets of remolded loess samples, each set containing three samples. Samples within each sample set were prepared in such a way that they would have identical WDD values but exhibit a range of uniformity degrees. The permeability was carefully determined for each sample using a constant head permeability test, and sample uniformity was assessed on the duplicate sample by carrying out detailed measurements of dry density. In this way the relationship between uniformity and permeability could be tested and assessed. Finally, mathematical equations were developed based on Darcy’s Law and Kozeny-Carman equation to provide verification for the relationships observed.

## Materials and methods

### Materials

**Figure 1:**
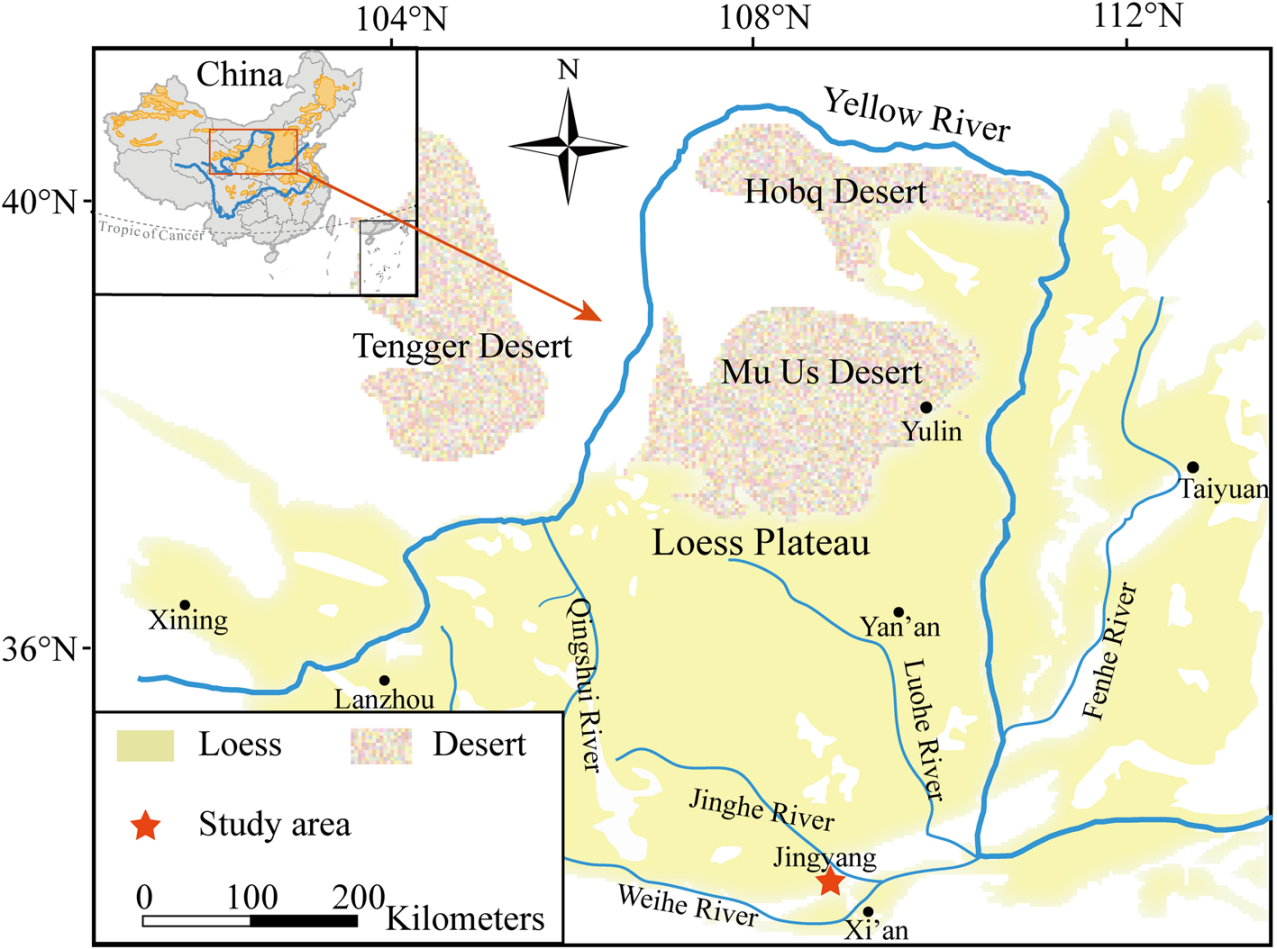
Location of the sampling site within China (inset).

The soil at the sampling site is Q_3_ loess (known as Malan loess in China), which belongs to the Late Pleistocene epoch and is also a common material for local constructions 1. The deposit varies in thickness from a few meters to over 50 m^30^. The sample was collected on the south bank of the Jinghe River, JingYang county, Shaanxi province (Fig. 1). After returning the sample to the laboratory, the loess was dried at 105 °C for eight hours, disaggregated, and passed through a 2-mm sieve before being thoroughly homogenized. In the next stage, the physical properties of the loess were determined using procedures specified in the Standard for Geotechnical Testing Method^31^. Additionally, the particle size distribution (PSD) of the soil was determined using a laser diffraction-based particle size analyzer.

**Figure 2:**
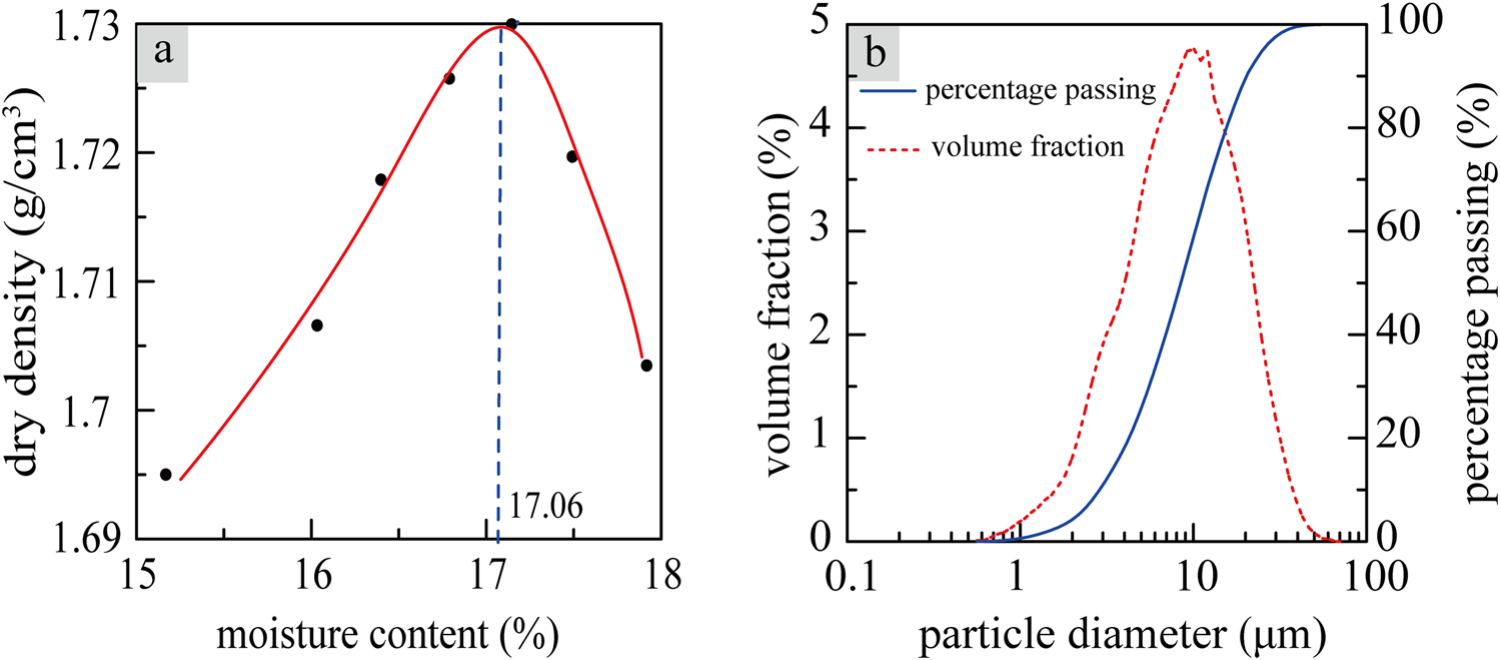
Determination of optimal moisture content by compaction (**a**). Particle size distribution (**b**).

The results of the analysis are shown in Table 1 and on Fig. 2. The PSD shows that the loess consists mainly of silt particles (i.e. 0.005–0.075 mm) with a fraction of 74.31%, sand particles (i.e. > 0.075 mm) with a fraction of 0.74%, clay particles (i.e. < 0.005 mm) with a fraction of 24.95%.

**Table 1:** The basic properties of the Malan loess.

Maximum dry density (g/cm^3)^	Optimal moisture content/%	Plastic limit /%	Liquid limit/%	Specific gravity	Specific surface area (m^2^/g)
1.73	17.06	17.67	32.19	2.71	59.4

The specific gravity and specific surface area of the loess were tested to be 2.71 and 59.4 m^2^/g, respectively, while the maximum dry density and optimal moisture content (as a mass percentage) were determined to be 1.73 g/cm^3^ and 17.06%, respectively. The loess sample revealed a liquid limit of 32.19% and a plastic limit of 17.67%. According to standard soil classifications, the loess can be characterized as a low plasticity silt. With respect to the Unified Soil Classification System, the loess is a low liquid limit clay (CLY).

### Sample preparation

To test the relationship between uniformity and permeability, three sets of loess samples were prepared in duplicate, each with a different value of whole dry density (WDD) (1.3 g/cm^3^, 1.4 g/cm^3^, and 1.5 g/cm^3^). Each set contained three samples. Samples were prepared using the well-established, moist tamping technique, a method that is relatively easy to use and provides good control over sample density.

To prepare the loess for tamping, the homogenized loess was sprayed with deionized water until an optimal moisture of approximately 17% (as a mass percentage) was achieved. The material was then remixed to achieve uniformity of color and texture. Finally, in order to reach moisture equilibrium, the soil sample was sealed in an airtight bag for at least 24 h.

**Figure 3:**
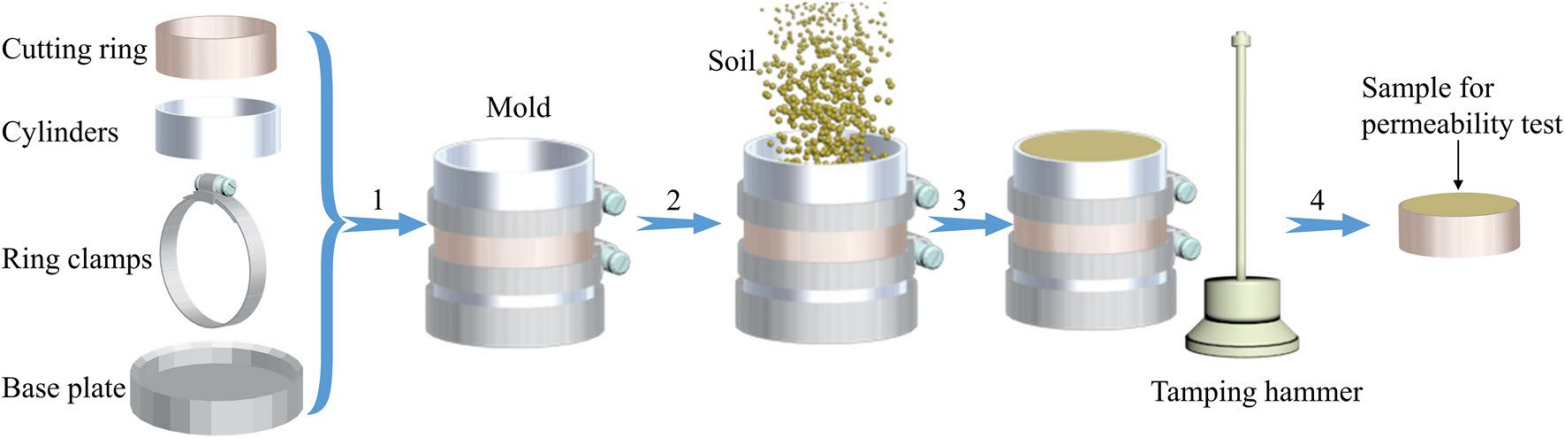
The soil samples remolding process (1. Connecting the cutting ring and split molds with ring clamps. 2. Installing mold on the base plate, and then adding soil to the mold. 3. Compacting soil with tamping hammer. 4. Removing the cutting ring from the mold).

The tamping equipment and procedures were designed to produce test samples of 61.8 mm in diameter and 40 mm in height, the dimensions required by the permeameter used to conduct the permeability test. It began with the construction of a three-part mold, 61.8 mm in diameter (Fig. 3) comprising a pre-weighed cutting ring of 40 mm high (containing the eventual sample) positioned between two stainless steel cylinders of the same diameter but with three possible heights for the lowermost cylinder (5, 10 and 30 mm). The reasons for these 3 heights are explained below. The mold was held together using ring clamps tightened at each seam. The mold was set vertically on a solid base plate.

Prior to sample preparation, a thin layer of petroleum jelly was applied to the inner wall of the mold to avoid potential wall-effects^32^. Then, a known mass of soil was added (pre-calculated based on the target WDD of the soil) and compacted using a tamping hammer. The tamping hammer weight is 0.7 kg and drop height is 300 mm. After tamping, the cutting ring containing the sample was carefully dismantled and any excess soil was removed from its top and bottom using a sharp knife. The cutting ring was then reweighed to calculate the WDD of the sample. Samples that did not meet exacting criteria (target WDD ± 0.0001 g/cm^3^) were rejected and the processes repeated until compliance was achieved.

Given that the purpose of the work was to test the relationship between permeability and uniformity, it was important to ensure that the three samples collected within each sample set of known WDD (1.3g/cm^3^, 1.4 g/cm^3^, and 1.5 g/cm^3^) exhibited different degrees of uniformity. This was achieved by i) ensuring that each 40 mm thick sample was collected at a different distance from the mold base plate (either 5, 10 or 30 mm), and ii) using two possible final compaction heights (80mm or 100mm). Thus, even though samples within each set had identical values of WDD (as confirmed by measurement), their experience to tamping would have been different as a function of sample depth and final compaction height i.e. it was anticipated, albeit unconfirmed at this stage, that the three samples within each set would display different degrees of uniformity.

Table 2 summarizes the preparation characteristics of each sample. The three sample sets are referred to as A, B and C with WDD values of 1.3g/cm^3^, 1.4 g/cm^3^, and 1.5 g/cm^3^, respectively. Within each set, the subscripts 1, 2 and 3 refer to samples that were collected at increasing distances from the baseplate (i.e. 5, 10 and 30 mm). In all cases, the thickness of the sample used to perform permeability determinations was 40 mm (the height of the cutting ring). However, final compaction heights for samples with subscripts 1 and 2 were 80 mm, while for subscript 3, the height was 100 mm.

**Table 2:** Detailed information for the preparation of each sample.

Number	Compaction height (mm)	Initial dry density (g/cm^3^)	Moisture content (%)	Tamping strikes	Sampling interval (with respect to base)	Whole dry density of samples (g/cm^3^)
A_1_	80	1.395	17	20	5–45 mm	1.300
A_2_	80	1.39	17	18	10–50 mm	1.300
A_3_	100	1.36	17	35	30–70 mm	1.300
B_1_	80	1.495	17	65	5–45 mm	1.400
B_2_	80	1.49	17	62	10–50 mm	1.400
B_3_	100	1.46	17	70	30–70 mm	1.400
C_1_	80	1.595	17	95	5–45 mm	1.500
C_2_	80	1.59	17	89	10–50 mm	1.500
C_3_	100	1.57	17	164	30–70 mm	1.500

### Determination of permeability

**Figure 4:**
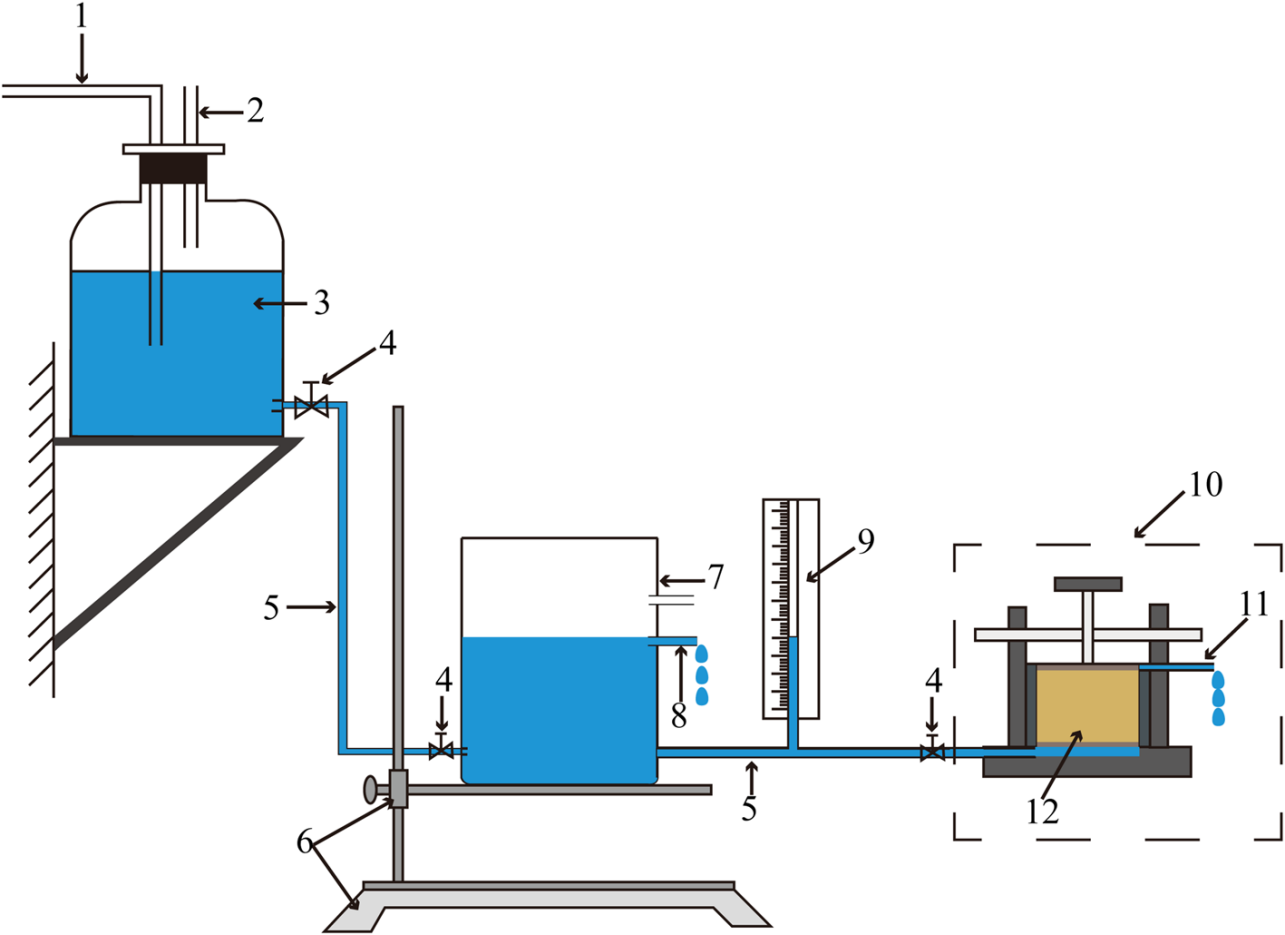
Schematic diagram of the permeability test setup (1. Water source; 2. Vent; 3. Water supply bottle; 4. Clamp valve; 5. Silicone tube; 6. Lifting table; 7. Water tank; 8. Drainage duct; 9. Piezometer tube; 10. Permeameter; 11. Outlet; 12. Loess sample).

*K* is the key hydraulic parameter of soil to reflect soil permeability. *K* was determined for each of the nine samples (three samples in each of three sets) by conducting a constant head permeability test. This method has been widely used to determine soil permeability^33^. The experimental set up is shown in Fig. 4. Silicone tubes were used to connect the two water reservoirs with the permeameter, which was installed vertically on a horizontal a platform. Saturation via falling head method was used for sample saturation to avoid the damage to soil structure in the process of saturation. Firstly, the samples were saturated slowly with the deionized water level rising at a rate of 1 cm/12 h for thoroughly removing air affecting the experimental results^34^. The level of water raised 4 times was defined as sample saturated. The hydraulic gradient used in the steady state test was set at the relatively low value of 3 to avoid the risk of soil scouring.

*K* was calculated according to a form of the Darcy equation:

1 $$ K = \frac{Q}{A \cdot i \cdot t} $$

where, *K* is the hydraulic conductivity (m/d); *Q* is the volume of water discharged (m^3^) in time* t* (d); *A* is the cross-sectional area of the sample (m^2^); *i* is the hydraulic gradient.

*K* is temperature dependent since temperature can affect both the density and viscosity of the water. In practice, the role of density change is negligible for small temperature variations; however, changes in viscosity due to temperature can have a measurable impact on *K* determinations. For reporting purposes, values of *K* determined during the tests were converted into *K*_20_ at 20°C (standard temperature) according to the equation^35^:

2 $$ K_{20} = K\frac{\eta }{{\eta_{20} }} $$

where, $K_{20}$ is the hydraulic conductivity at the standard temperature (20 °C); and *η* and *η*_20_ are the coefficients of dynamic viscosity at the test temperature and at 20 °C, respectively.

### Sample uniformity analysis

The dry density should be the same in all parts of a homogeneous loess sample, but different in heterogeneous sample. The degree of uniformity can be analyzed by measuring the change of dry density inside the loess sample. Sample uniformity analysis was performed on the duplicate sample set as the sample set used for permeability test could not be reliably re-used. For each of the nine samples, uniformity analysis was performed for the entire compacted column (i.e. from the base of the mold below the sample cutting ring to the top of the column above the sample cutting ring—a total of 80 mm for samples with subscripts 1 and 2, and 100 mm for samples with subscript 3). To achieve this, the soil compacted soil columns were slowly and carefully extruded from the mold and layered with a sharp knife from base to surface at measured intervals of between 5 and 10mm. Each layer was then weighed to calculate the dry density. The results are presented and discussed in Section "Results and discussion" below.

**Figure 5:**
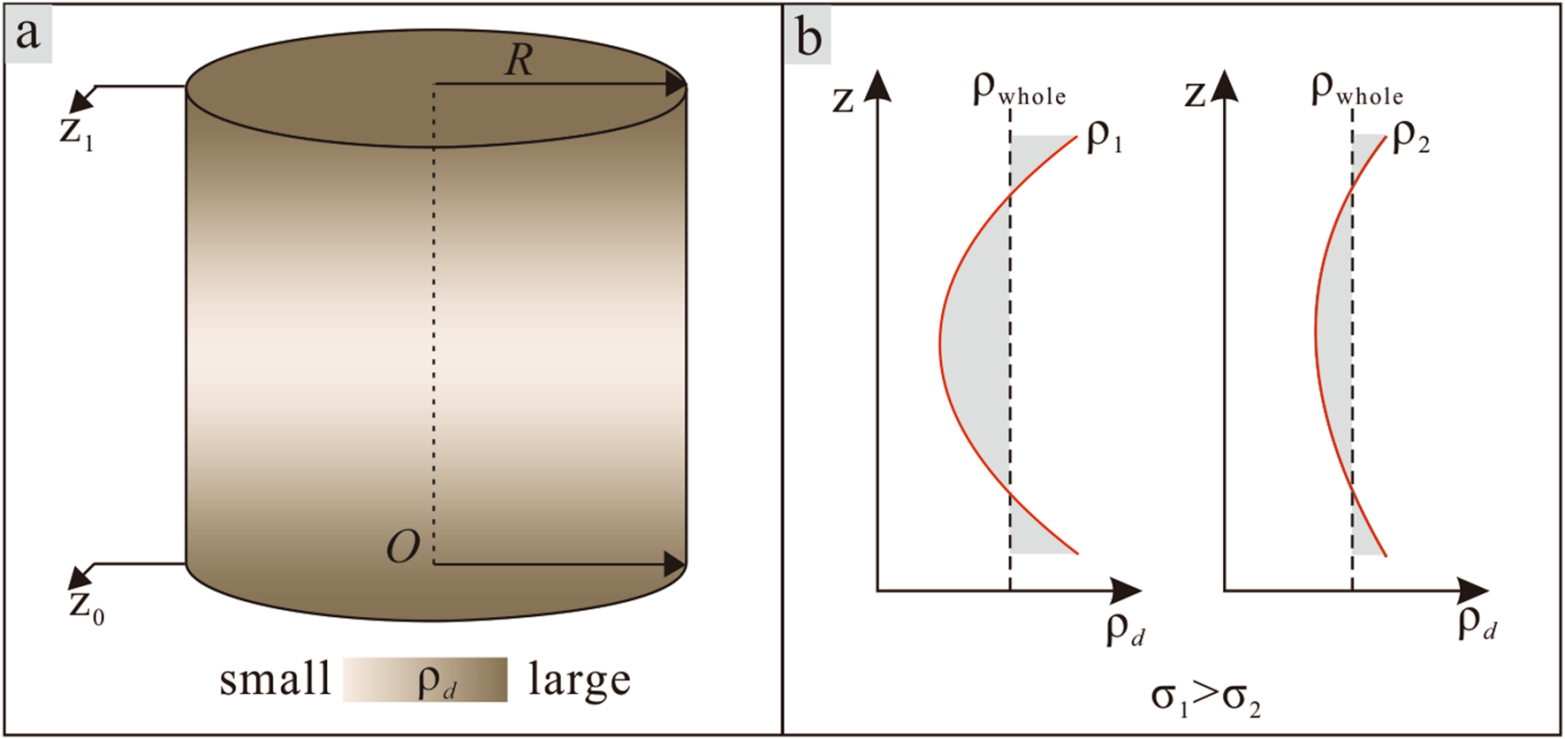
Schematic diagram of heterogeneous sample and calculation of variance of soil sample.

Variance ($\sigma^{2}$) is a measure of how far a set of observations deviates from its average value. Thus, it represents a measure of uniformity degree. In this study, the variance of dry density was used as an index of sample uniformity. Variance was calculated according to the equation:

3 $$ \sigma^{2} = \int_{{z_{0} }}^{{z_{1} }} {\left( {\rho (z) - \rho_{whole} } \right)^{2} dz} $$

where, *ρ*(*z*) is variation of density as a function of distance* z* from the base plate; *ρ*_*whole*_ is average dry density of the WDD; *z*_0_ and *z*_1_ represent the top and bottom positions of the sample such that *z*_1_-*z*_0_ = 40mm (the sample thickness) (as show in Fig. 5a). It is not difficult to conclude that the uniformity degree of sample is high, the dry density in soil sample is close to it average value, and then the variance is small; on the contrary, the uniformity degree is low, the is large (Fig. 5b).

## Results and discussion

### Analysis of the experimental data

#### Variations of dry density

**Figure 6:**
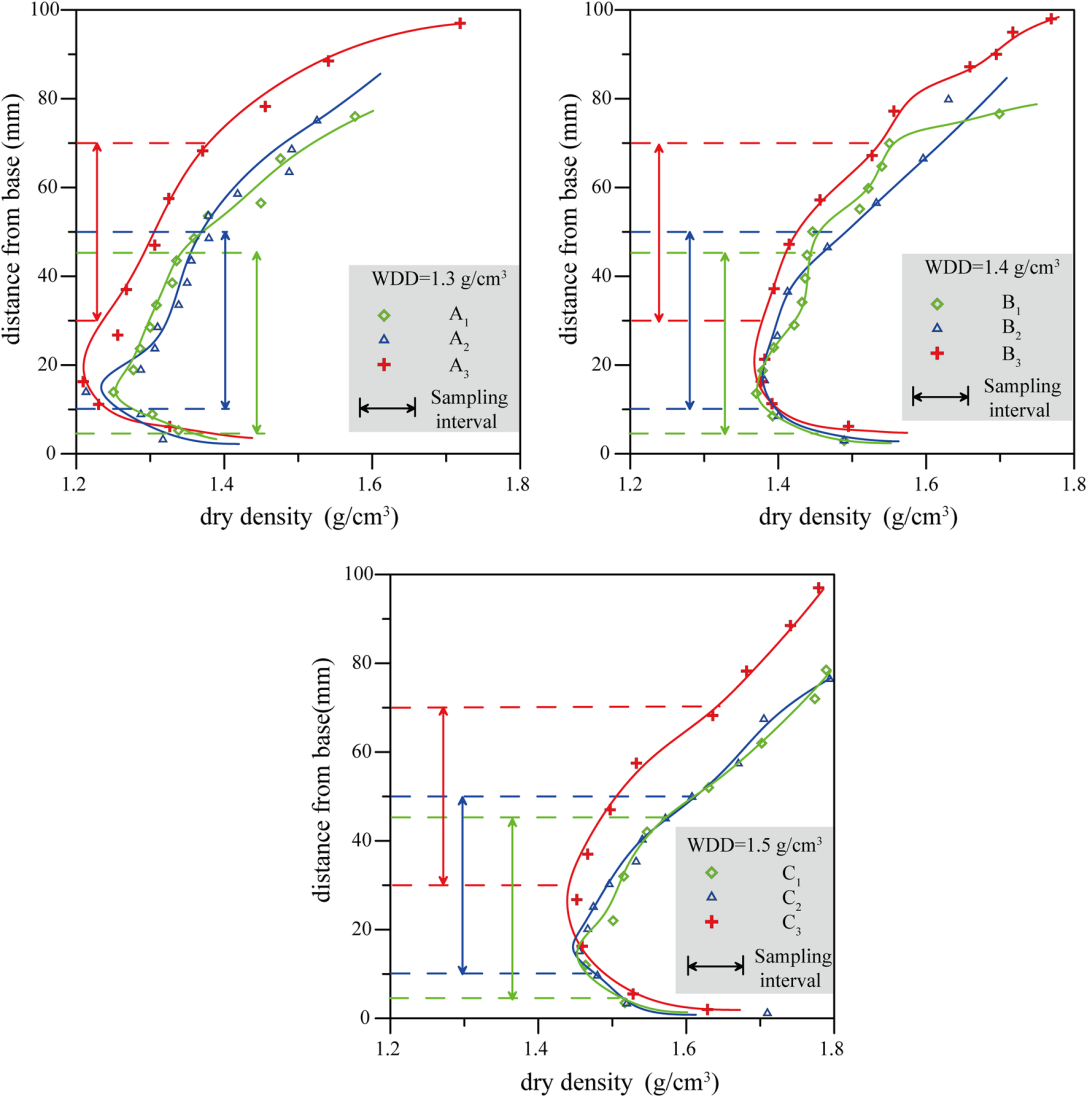
Uniformity as reflected by dry density changes in the compacted loess columns. Sampling intervals shown refer to the 40 mm thick sample zone (or “cutting ring”) used for permeability test.

Details of the dry density variations are provided in Table 3. They clearly demonstrate that the task of preparing three samples with varying degrees of uniformity within each sample set of known WDD (1.3 g/cm^3^, 1.4 g/cm^3^, and 1.5 g/cm^3^) was achieved successfully. The data are plotted in Fig. 6. Variations in dry density show a similar trend for all three sample sets (A, B and C) (Fig. 6). The dry density decreases rapidly from surface downwards to the depth of approximately 20 to 30 mm. Then, there is a gradual decline in dry density downwards until a minimum value is reached approximately 10–20 mm above the sample base. Thereafter, dry density increases sharply. These findings are similar to those of Marketos and Bolton^36^ and Soriano et al.^37^.

**Table 3:** Range of dry density for the nine simples.

Number	Range of dry density (g/cm^3^)	Sampling interval (mm)	WDD (g/cm^3^)	Range of dry density in cutting ring (g/cm^3^)
A_1_	1.48–1.577	5–45	1.3	1.248–1.341
A_2_	1.213–1.565	10–50	1.3	1.213–1.379
A_3_	1.209–1.718	30–70	1.3	1.209–1.425
B_1_	1.370–1.698	5–45	1.4	1.370–1.439
B_2_	1.381–1.671	10–50	1.4	1.381–1.475
B_3_	1.377–1.769	30–70	1.4	1.377–1.532
C_1_	1.463–1.789	5–45	1.5	1.551–1.463
C_2_	1..455–1.794	10–50	1.5	1.571–1.455
C_3_	1.452–1.799	30–70	1.5	1.622–1.452

**Figure 7:**
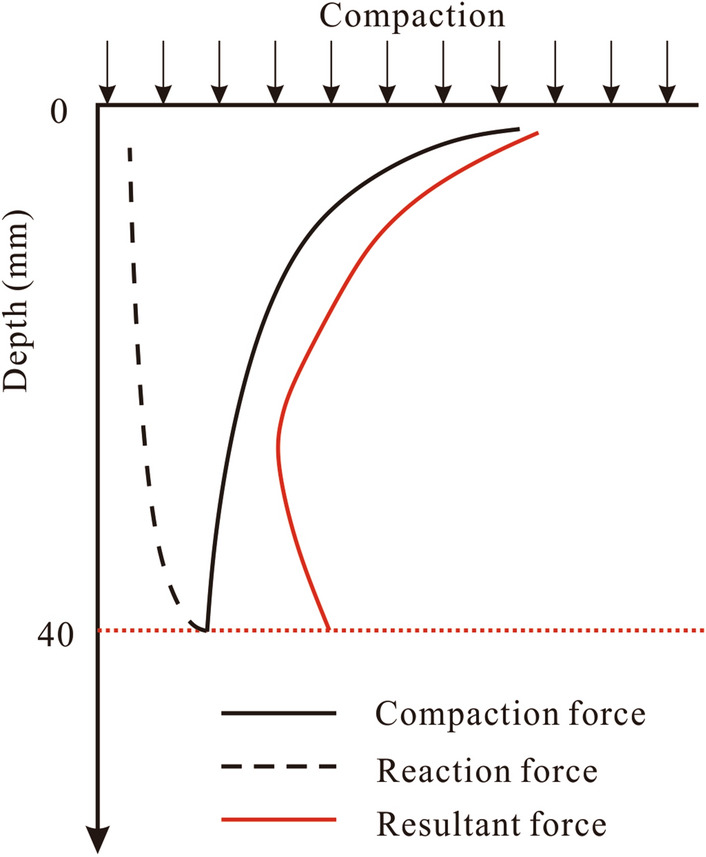
The schematic vertical stress distribution in loess column under a circular footing (modified after Ishibashi & Hazarika (2015))^38^.

During sample preparation, regardless of the technique used, an external force is needed to compact the sample into the required state (Fig. 7). This force is applied uniformly across the surface of the sample and will decrease in magnitude gradually from the surface to the base in a vertical direction^38^. Summarizing a number of experimental experiences, Ménard and Broise^39^ proposed an empirical equation as showed below would provide an estimate of the influence depth:

4 $$ Z = \sqrt {GH} $$

where, *Z* is the influence depth (m),* G* is the weight of hammer (kg), and *H* is the drop height (m).

According to the hammer weight (0.7 kg) and drop height (300 mm) used in this study, the influence depth was approximately 144 mm, far greater than the sample height (40 mm). During sample preparation, the hammer produces a compaction force on the sample surface and transmit through the loess sample from the top to bottom. The compaction force compacts the loess sample firstly. Then, there is residual force at the base. Residual force and base contact produce elastic collision to form equal, reverse reaction force (black dotted line in Fig. 7). Reaction force transfers from base to top and decreases gradually. The reaction force compacts the loess sample secondly. Both the forces have compaction effect on loess sample, therefore, the two forces are superposed by considering the compaction effect. The net product is a resultant force shown by the red line in Fig. 7. This force is highest at the surface of the sample and gradually reduces with depth, reaching a minimum value just above the sample base. Thereafter it increases sharply until the base plate is encountered. Variations in the resultant force explain the vertical changes in dry density observed during experimentation (Fig. 6).

#### Analysis of sample uniformity

To characterize degree of sample uniformity, the dry density variations were fitted with quartic functions. The fitting lines are included on Fig. 6 and the functions derived are shown in Table 4. In all cases, the quality of the fit as determined through correlation coefficients was found to be greater than 90%. In all nine samples, discrepancies between observed and calculated values of dry density never exceeded 0.048 g/cm^3^.

**Table 4:** The results of dry density fitting curve.

Number	Fitting functions of the loess columns	Correlation coefficient/*R*^2^	Dry density error for the entire loess column (g/cm^3^)	Variance (*σ*^2^) of the loess sample	Dry density error for the section containing the loess sample (g/cm^3^)
A_1_	y = 0.0006z^4^ − 0.0095z^3^ + 0.0592z^2^ − 0.1173z + 1.3404	0.9356	0.016	0.00566	0.01
A_2_	y = 0.0006z^4^ − 0.0099z^3^ + 0.0673z^2^ − 0.1637z + 1.3998	0.9696	0.017	0.00612	0.01
A_3_	y = 0.0006z^4^ − 0.012z^3^ + 0.0862z^2^ − 0.2257z + 1.4144	0.9872	0.016	0.00705	0.01
B_1_	y = 0.0012z^4^ − 0.02z^3^ + 0.1146z^2^ − 0.2409z + 1.5407	0.962	0.048	0.00234	0.01
B_2_	y = 0.0001z^4^ − 0.0047z^3^ + 0.0503z^2^ − 0.1549z + 1.5192	0.984	0.036	0.00382	0.01
B_3_	y = 0.0002z^4^ − 0.0056z^3^ + 0.054z^2^ − 0.1866z + 1.5704	0.9872	0.021	0.00468	0.01
C_1_	y = − 0.0002z^4^ + 0.0013z^3^ + 0.0112z^2^ − 0.0479z + 1.5251	0.9907	0.019	0.00272	0.01
C_2_	y = 0.0004z^4^ − 0.0079z^3^ + 0.0592z^2^ − 0.139z + 1.5608	0.9939	0.028	0.00912	0.01
C_3_	y = 0.0001z^4^ − 0.0039z^3^ + 0.0461z^2^ − 0.172z + 1.6383	0.9818	0.029	0.0104	0.02

Also included in Table 4 is the variance of the sample density for each of the loess samples. As indicated by the variance values shown in Table 4, all nine samples exhibited some lack of uniformity (*σ*^2^ > 0). Within each sample set (A, B and C), degree of sample uniformity is highest for samples taken closest to the base of the compacted column (sample interval 5–45 mm—subscript 1) and is lowest for the samples taken close to the top of the compacted column (sample interval 30–70 mm—subscript 3). Although this suggests that degree of sample uniformity is best achieved by taking samples close to the bottom of the tamping column, reference to Fig. 6 suggests that the very base of the column should be avoided due to the rapid increases in density that occur as the base plate is approached.

#### Relationship between uniformity and permeability

**Figure 8:**
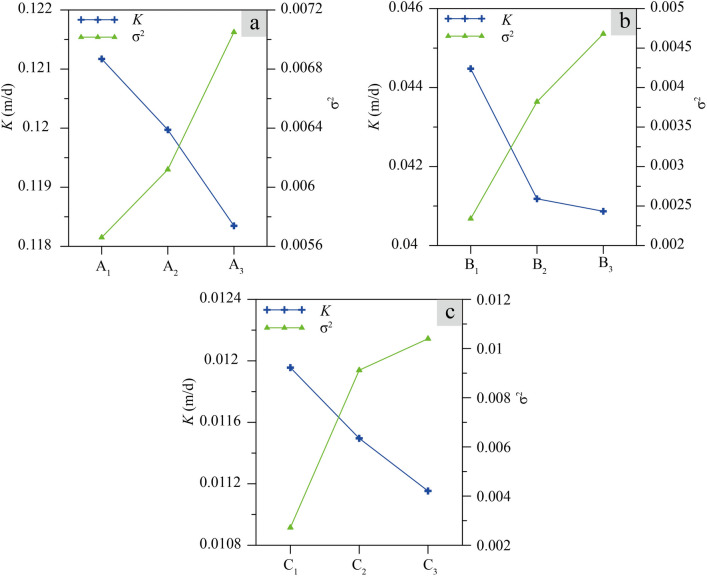
The relationship between variance ($\sigma^{2}$) and hydraulic conductivity (*K*) for the nine loess samples. Whole dry densities for (**a**–**c**) are 1.3 g/cm^3^, 1.4 g/cm^3^ and 1.5 g/cm^3^, respectively.

Values of *K* obtained for the nine samples are shown in Fig. 8 where they are compared to degree of sample uniformity as represented by variance ($\sigma^{2}$). For all three sample sets, *K* is negatively correlated with $\sigma^{2}$ meaning it is positively correlated with uniformity degree. In other words, the more uniformity of loess sample, the greater its permeability.

The relationship can be anticipated because *K* is dependent on the loess pore structure (pore size, pore size distribution and pore connectivity)^20,29,40,41^, and spatial variations in pore structure are primary causes of soil sample heterogeneity^42^. Thus, while samples may have identical values of WDD i.e. identical values of total porosity, the pore sizes and *K* between those pores may vary throughout each sample resulting in different degrees of uniformity^43^. As suggested by Long and Witherspoon^44^, *K* will decrease with reducing pore connectivity for samples having the same porosity. A similar conclusion was drawn by Aydogan and Hyttinen^43^ based on his examination of six different types of microstructure in samples of the same porosity. It would seem that poor uniformity degree (and high variance of dry density) are associated with a reduction pore connectivity, such that high degree of uniformity will give rise to increased pore connectivity and higher values of *K*.

### Mathematical model

#### Theoretical deduction

Experimental results show that loess samples with high degree of uniformity have bigger values of *K*. However, the results only qualitatively give the relationship between uniformity and permeability without strict theoretical verification. Here this finding is investigated on a theoretical basis through a mathematical examination of the relationship between degree of sample uniformity and *K*.

**Figure 9:**
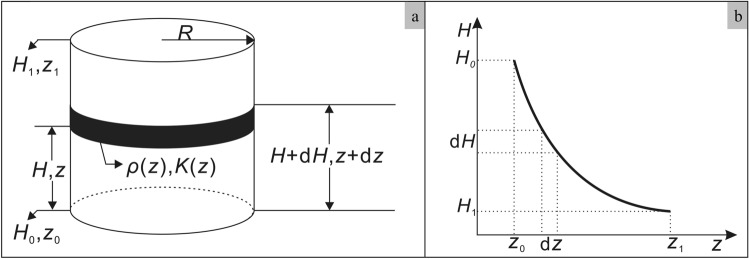
The schematic diagram of the loess sample (**a**) and the process of head loss during permeability test (**b**).

It can be assumed that dry density changes in the horizontal direction are negligible i.e. that the dry density and *K* the of loess sample change only in the vertical direction (Fig. 9a).

The vertical distribution of water head during the permeability test is shown schematically in Fig. 9b. Where, *ρ*(*z*) is the dry density, *K*(*z*) is hydraulic conductivity, *R* is the radius of loess sample, then according to Darcy’s Law:

5 $$ V = - K(z)\frac{dH}{{dz}} $$

or

6 $$ - dH = \frac{V}{K(z)}dz $$

where, *H*_0_ is the head at *z*_0_ (water inlet end); *H*_1_ is the head at *z*_1_ (water outlet end); and *V* is the specific discharge (volumetric flow rate per unit area).

Equation (6) can be integrated in the vertical direction to give:

7 $$ \int_{{H_{0} }}^{{H_{1} }} { - dH = \int_{{z_{0} }}^{{z_{1} }} {\frac{V}{K(Z)}dz} } $$

Given $\Delta H = H_{0} - H_{1}$, Eq. (7) can be rewritten as:

8 $$ \Delta H = V\int_{{z_{0} }}^{{z_{1} }} {\frac{1}{K(Z)}dz} $$

The Kozeny-Carman equation is widely used to predict the *K* of porous media^45^. In the Kozeny-Carman equation, the *K* can be expressed as

9 $$ K = \frac{{C_{0} }}{{S_{s}^{2} }}\frac{g}{{\mu_{w} \rho_{w} \rho_{s}^{2} }}\frac{{\varphi^{3} }}{{(1 - \varphi )^{2} }} $$

where $\varphi$ is porosity of loess sample, which is dimensionless; *C*_0_ is a dimensionless constant; *g* is the gravitational constant; *S*_*s*_ is the specific surface area of particles; *ρ*_*s*_ is the particle density of soil; *ρ*_*w*_ is density of water and *μ*_*w*_ is dynamic viscosity of water. $\varphi$ is directly related to *ρ*_*d*_, which can be expressed as

10 $$ \varphi = 1 - \frac{{\rho_{d} }}{{\rho_{s} }} $$

Therefore, the *K*(*z*) can be expressed as

11 $$ K(z) = \frac{{C_{0} }}{{S_{s}^{2} }}\frac{g}{{\mu_{w} \rho_{w} \rho_{s}^{3} }}\frac{{(\rho_{s} - \rho_{d} (z))^{3} }}{{(\rho_{d} (z))^{2} }} $$

In Eq. (11), *g*, *μ*_*w*_ and *ρ*_*w*_ are constants independent of soil sample. *ρ*_*s*_ and *S*_*s*_ are inherent properties of soil that only depend on the soil type. In this study, the same soil was used for remolding loess sample. Therefore, *ρ*_*s*_ and *S*_*s*_ can be regarded as constants in different samples.

By substituting Eq. (11) into Eq. (8), the expression can be rewritten as:

12 $$ \Delta H = \frac{{VS_{s}^{2} \mu_{w} \rho_{w} \rho_{s}^{3} }}{{C_{0} g}}\int_{{z_{0} }}^{{z_{1} }} {\frac{{\left( {\rho_{d} (z)} \right)^{2} }}{{\left( {\rho_{s} - \rho_{d} (z)} \right)^{3} }}} dz $$

The *K* of the whole sample is defined as the average hydraulic conductivity ($\overline{K}$) in this study. $\overline{K}$ can be expressed as

13 $$ \overline{K} = \frac{VL}{{\Delta H}} $$

where *L* is the total height of soil sample. By substituting Eq. (11) into Eq. (12), the relationship between dry density changes and $\overline{K}$ can be obtained, and the expression can be rewritten as:

14 $$ \overline{K} = \frac{C}{{\int_{{z_{0} }}^{{z_{1} }} {\frac{{\left( {\rho_{d} (z)} \right)^{2} }}{{\left( {\rho_{s} - \rho_{d} (z)} \right)^{3} }}} dz}} $$

where $C = \frac{{C_{0} gL}}{{S_{s}^{2} \mu_{w} \rho_{w} \rho_{s}^{3} }}$ is constant.

Equation (14) indicates that $\overline{K}$ and $\int_{{z_{0} }}^{{z_{1} }} {\frac{{\left( {\rho_{d} (z)} \right)^{2} }}{{\left( {\rho_{s} - \rho_{d} (z)} \right)^{3} }}} dz$ has inversely proportion. If the dry density function ($\rho_{d} (z)$) can be determined for the soil sample, by solving the integral term of Eq. (13), the $\overline{K}$ with different dry density changes can be obtained. Based on $\rho_{d} (z)$, degree of sample uniformity can be calculated using Eq. (4). Therefore, the relationship between degree of sample uniformity and $\overline{K}$ can be analyzed by Eqs. (14) and (4).

#### Verification of the experimental results

The dry density changes of loess samples in this study were fitted with quartic functions (Table 4). Each sample set should have the same WDD. The WDD for the section containing the loess sample of each functions needs to be verified. Given that the WDD ($\rho_{whole}$) of each sample set is fixed, $\rho_{d} (z)$ must satisfy the following constraint:

15 $$ \int_{{z_{0} }}^{{z_{1} }} {\rho_{d} (z)dz = } \int_{{z_{0} }}^{{z_{1} }} {\rho_{whole} dz} = M $$

where, *M* represents the mass of the soil sample at each WDD.

Dry density error between the calculated value and the set value is shown in Table 4. For the section containing the cutting ring (the sample) the maximum error never exceeded 0.02 g/cm^3^. It can be considered that the quartic function meets the requirement of the same WDD in each set.

**Figure 10:**
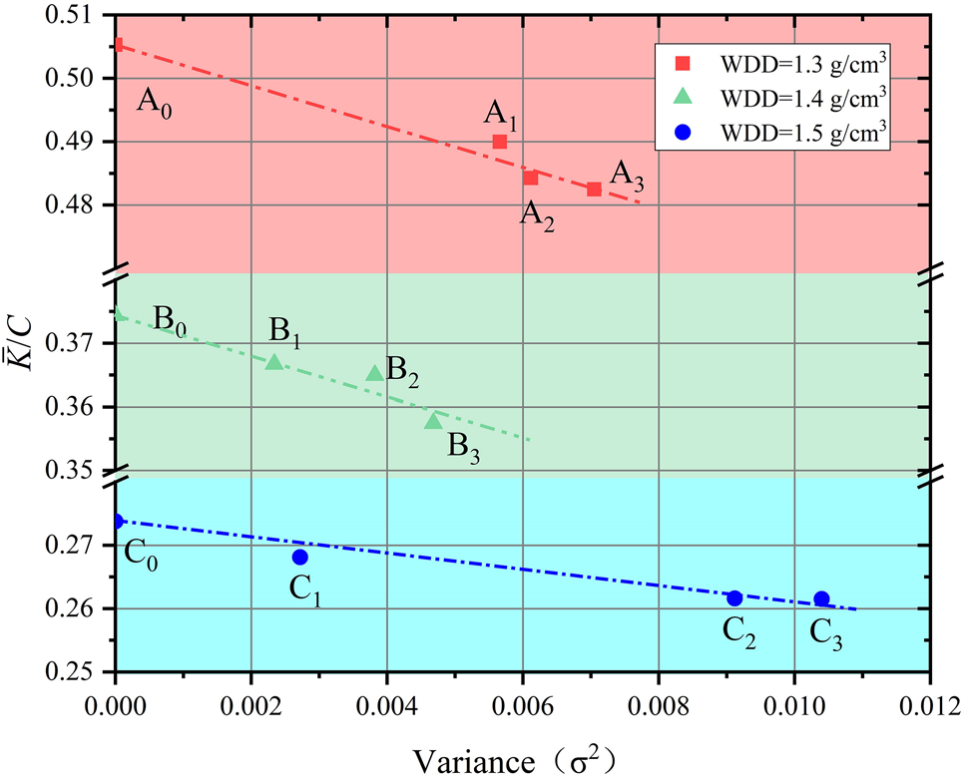
The relationship between variance (${\sigma }^{2}$) and $\overline{K}$ for three uniform samples and the nine test samples.

The $\sigma^{2}$ and $\frac{{\overline{K} }}{C}$ of the nine loess samples were calculated using Eqs. (3) and (14) and are plotted in Fig. 10. For each set of samples, a homogeneous sample with constant density (A_0_:1.3 g/cm^3^, B_0_:1.4 g/cm^3^, C_0_:1.5 g/cm^3^) was included (perfectly uniform with $\sigma^{2} = 0$). In each case, a negative correlation is observed between $\sigma^{2}$ and $\frac{{\overline{K} }}{C}$. Results of this analysis show that $\overline{K}$ is biggest for the homogeneous sample at each WDD (perfectly uniform with $\sigma^{2} = 0$). As $\sigma^{2}$ increases, $\overline{K}$ decreases (Fig. 10). This implies that *K* is positively correlated with degree of sample uniformity, a result that is consistent with the results of the permeability test (Fig. 7). For samples having the same WDD, the sample with the largest *K* is always the best uniform degree.

### Engineering significance

The implementation of mega geotechnical project in loess region generates the study of loess geotechnical engineering to a hot spot. For example, the land creation project creates 78.5 km^2^ for land development in Yan’an city through large-scale and high-fill remolded loess^46^. In engineering construction, to reduce engineering disasters, it is necessary to reduce the permeability of remolded loess by compaction^41^. Li, et al.^46^ found that half of the remolded loess was less than the required compaction for the construction in some high-embankment loess in Yan’an, indicating that heterogeneous remolded soil would inevitably be caused in the process of engineering construction. Therefore, detailed understanding of the influence of uniformity on permeability can provide a basis for the design and construction of high fill engineering in loess region and offer a reference for the future construction in the Loess Plateau.

**Figure 11:**
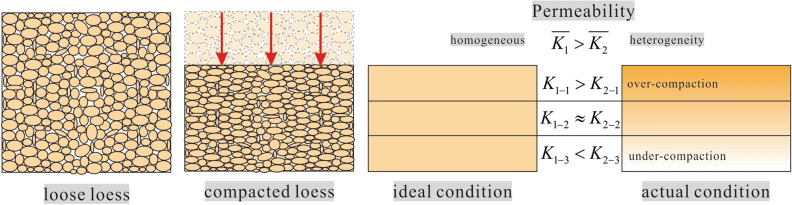
Schematic diagram of loess compaction and its permeability.

The permeability of heterogeneity loess is lower than homogeneous loess, but it does not mean that it is effective to reduce permeability of remolded loess by reducing uniformity. The heterogeneity inevitably leads to over-compaction and under-compaction areas in remolded loess (Fig. 11). Although the permeability of whole sample meets construction requirements, due to the heterogeneity of remolded loess, permeability may be underestimated, especially in the under-compaction area (Fig. 11). The loess in under-compaction area is lower than the density requirement, resulting in strong permeability in this area. Pore structure of low density loess is unstable and prone to collapsible and deformation^41^. Pu, et al.^47^ observed the land subsidence of remolded loess in Yan’an and found that there was a large settlement amount and high settlement ratio in the heterogeneous area. The results show that the under-compaction area of heterogeneous loess has serious disaster risk. Therefore, in engineering design and construction, reasonable compaction technology and compaction scheme should be adopted to improve the uniformity degree of remolded loess while reducing the permeability.

The uniformity will affect the permeability on loess, which provokes a question worth thinking, namely, whether the physical and mechanical parameters measured by heterogeneous samples in the laboratory can fully reflect the real condition? Due to the same compaction mechanism, heterogeneous loess is inevitably produced both in field construction and laboratory remodeling. Kuerbis and Vaid^48^ highlighted the need for samples used in laboratory tests to meet high degrees of uniformity. This implies that sample uniformity should be assessed prior to any other test, with those samples failing to meet strict uniformity standards rejected from further analysis. Studies have shown that the degree of soil samples uniformity will affect the results of shear tests^49^, unconfined and triaxial tests^50^, water-holding tests^51^ and testing for soil–water characteristics^52^. However, there is no universally method to measure uniformity degree of soil. At present, thermal sensor, X-ray, gamma-ray, optical analysis and measured dry density by layer have been used to analyze the uniformity of sample^15,26,53^. Unfortunately, existing methods for analyzing sample uniformity are difficult, time-consuming and uneconomic. The result of this paper provides a new idea for testing the degree of sample uniformity, that is, *K* can be used with confidence as an index of sample uniformity. According to the established relationship between permeability and sample uniformity, the soil sample with largest *K* has the best uniformity degree in the same WDD. This method ensures that the permeability is not underestimated while selecting loess sample with best uniformity degree. Permeability is required to be measured in almost all geotechnical tests. It can not only overcome the shortcomings of previous methods, but also not add too much extra work.

## Summary and conclusions

In this study, the dry density distribution and *K* were tested respectively. The variation characteristics of the dry density in remolded less were analyzed firstly, and more importantly, the relationship between sample uniformity and *K* was clarified. The difference of sample preparation conditions will affect the dry density distribution, resulting in inconsistent of sample uniformity. The laboratory tests on loess samples show that values of *K* increase with increasing degree of sample uniformity, a finding that was verified through mathematical analysis. The permeability of heterogeneous loess is less than homogeneous loess, but its permeability is often underestimated. High degree of loess uniformity should be ensured when reducing the permeability of remolded loess by compaction.

Loess sample uniformity can seriously influence the results of laboratory tests, but is rarely measured, as it can be a difficult and time-consuming process. Consequently, the reliability of laboratory test data is rarely known. The recognition of *K* as a sensitive measure of sample uniformity solves this dilemma. The measurement of *K* is very easy to undertake and can be added to laboratory testing routines with little inconvenience and only minor additional cost.

To date, laboratory testing of the technique has been confined to loess soils. However, the rigorous mathematical analysis undertaken to verify the findings strongly suggests the relationship can be extended to all soil types with confidence. It should be noted that the study only considers dry density variations in the vertical direction but for most sample preparation techniques, this assumption is entirely appropriate. It should also be noted that this study did not consider the effect of fractures as the vast majority of remolded samples are not fractured. However, this is a topic that will be pursued in future investigations.

## Data Availability

The datasets used and analyzed during the current study available from the corresponding author on reasonable request.
